# Development of an instrument to measure the cultural competence of health care workers

**DOI:** 10.11606/s1518-8787.2020054001695

**Published:** 2020-03-12

**Authors:** Victor Pedrero, Margarita Bernales, Macarena Chepo, Jorge Manzi, Miguel Pérez, Paulina Fernández

**Affiliations:** I Universidad Andrés Bello Facultad de Enfermería Santiago Chile Universidad Andrés Bello . Facultad de Enfermería , Santiago , Chile; II Pontificia Universidad Católica de Chile Facultad de Medicina Escuela de Enfermería Santiago Chile Pontificia Universidad Católica de Chile . Facultad de Medicina , Escuela de Enfermería , Programa de Salud Global, Santiago , Chile; III Pontificia Universidad Católica de Chile Facultad de Ciencias Sociales Escuela de Psicología Santiago Chile Pontificia Universidad Católica de Chile . Facultad de Ciencias Sociales . Escuela de Psicología , Santiago , Chile; IV California State University Fresno College of Health and Human Services Department of Public Health California Estados Unidos California State University Fresno . College of Health and Human Services . Department of Public Health , California , Estados Unidos; V Psychologist. Independent consultant

**Keywords:** Cultural Competency, Culturally Competent Care, Health Knowledge, Attitudes, Practice, Validation Studies

## Abstract

**OBJECTIVE:**

To validate an instrument measuring the cultural competence in health care workers from Chile.

**METHODS:**

Using Sue & Sue’s theoretical model of cultural competence, we designed a scale, which was assessed by health care workers and experts. Subsequently, the scale was applied to a sample of 483 different health care workers, during 2018 in Santiago de Chile. The analysis included: exploratory and confirmatory factor analysis, estimation of reliability, and analysis of measurement bias. Finally, the level of cultural competence was calculated for every professional who participated in this study.

**RESULTS:**

The final scale include 14 items that are grouped into three dimensions concordant with the theoretical model: sensitivity to own prejudices, cultural knowledge, and skills to work in culturally diverse environments. This scale showed good fit in factor models, adequate reliability and lack of evidence of measurement bias. Regarding the performance of health care workers, sensitivity showed a lower level compared with the other dimensions evaluated.

**CONCLUSION:**

The scale for measuring the level of cultural competence in health care workers (EMCC-14) is a reliable instrument, with initial support for its validity, which can be used in the Chilean context. Additionally, the results of this study could guide some possible interventions in the health sector to strengthen the level of cultural competence.

## INTRODUCTION

Chile has progressively become a more diverse country as 4.4% of its population is composed of foreign immigrants ^1^ ; of these, 12.8% declare themselves to belong to an indigenous group ^1^ and 3.1% of men and 2.3% of women identify themselves with a gender different from their sex of birth ^2^ . In health care, Chile is committed to providing patient-centred health care ^3^ . It means, among other things, addressing the particular needs of different population groups and providing treatment free of discrimination. To achieve this, it is important to consider the role of cultural aspects in people’s relationship with the processes of health and disease ^3^ .

Culture corresponds to a system of shared conceptual frameworks and schemes used by the members of a group (or subgroup) to interpret reality and relate to the world around them, including their health ^4^ . When cultural aspects are not considered in health care, interactions between patients and professionals are less patient-centred, shorter, and less positive ^5^ . Cultural competence (CC) in health refers to the permanent reflection and questioning by the health care worker about how culture – of the professional and of the patient – impacts on the interaction with the users of the health system ^6^ . CC, includes a commitment to delivering services which take into account the patient’s beliefs and actions.

Although there are different definitions of CC, it is a multidimensional concept (includes at least three areas: sensitivity, skills and knowledge) and multilevel (involves both health professionals and health systems) ^7 , 8^ . Sue & Sue ^8^ propose a conceptual framework for CC that includes these two aspects, and four levels of cultural competence are considered: individual (health professionals), professional (professional practices), organizational (institutional practices and policies), and social (social policies). At each of these levels, the following should be considered: sensitivity to prejudices, knowledge of cultural aspects, and the ability to integrate these aspects into care.

One of the relevant characteristics of CC is that it can be trained ^9^ . However, its measurement remains a challenge, which makes it difficult to evaluate the effectiveness of interventions in this field ^9^ . There are several scales for measuring CC, but aspects associated with their validity ^10 , 11^ have been questioned. On the other hand, several instruments usually highlight the relevance of ethnic and racial aspects over other cultural encounters, which could make its use difficult in other contexts where these factors do not predominantly explain cultural diversity ^12^ .

This study aimed to validate a measurement instrument for CC in Chile.

## METHODS

The development of the Measurement Scale of CC in health care workers in Chile (EMCC-14) considered ^13^ : (i) item development, (ii) content validity study; (iii) internal structure validity analysis; (iii) reliability estimation and; (iv) bias analysis. The data collection took place in the city of Santiago de Chile during 2018.

### Development of Items

In addition to considering the components proposed by Sue & Sue ^8^ , we reviewed the literature concerning CC and measurement instruments previously published. The first version of the EMCC-14 had 31 items divided into three theoretical dimensions (sensitivity, knowledge and skills) ^9^ . The final version of the EMCC-14 has 14 items ( Table 1 ). Eight of these were adapted from previous instruments ^14 - 17^ and six were created by the authors.

**Table 1:** Cultural Competence Measurement Scale for Health Care Workers (EMCC-14), Santiago, Chile 2018.

Thinking about your usual clinical practice, for each question, indicate how much you agree or disagree.	Totally disagree	Disagree	Neither agree nor disagree	Agree	Totally Agree
	(1 point) (%)	(2 points) (%)	(3 points) (%)	(4 points) (%)	(5 points) (%)
1. I believe patients with different beliefs and customs have different expectations and/or needs in health care ^a^ .	7.1	10.2	9.6	38.1	35.0
2. I believe patients’ beliefs, values and customs affect their health ^b^ .	8.3	10.6	17.0	37.6	26.6
3. I believe my cultural context influences my attitudes and beliefs about other cultural groups ^c^ .	18.9	21.8	15.6	30.9	12.9
4. I am aware that my beliefs about patients influence the therapeutic recommendations I make to them.	20.8	23.1	18.3	28.5	9.2
5. Patient beliefs, values and customs should be appreciated in health care.	0.2	0.6	2.9	33.2	63.1
6. I believe that knowing more about patients’ beliefs and habits helps me to plan a more appropriate treatment.	0.8	1.0	7.9	36.4	53.8
7. I believe that each patient has his or her own concept of health and illness.	0.2	1.9	7.1	45.0	45.9
8. I believe the patient’s health problems must be understood within their cultural context (beliefs, values and customs) ^d^ .	0.2	1.1	6.6	48.2	44.0
9. I ask the patient and his/her family to express their expectations regarding health care ^b^ .	1.0	6.6	18.0	46.7	27.6
10. I am able to recognize potential barriers that different patients may face to accessing health services ^b^ .	0.4	2.1	7.0	61.9	28.5
11. I am able to set therapeutic goals and/or objectives considering the cultural context (beliefs and customs) of my patients and their needs ^c^ .	0.6	1.5	14.9	51.0	32.0
12. I record in the clinical file the data about beliefs and customs collected in the evaluation of the patient.	4.9	11.1	21.1	38.9	24.0
13. I make an effort to explain to the patient his/her medical treatment, even if he/she believes that the cause of his/her illness is supernatural.	0.2	1.1	5.9	44.4	48.4
14. I am aware of possible difficulties that may arise during health care due to cultural differences between the patient and me.	0.2	1.1	4.5	49.9	44.3

^a^ Items adapted from Cai et al. ^14^

^b^ Items adapted from Doorenbos et al. ^15^

^c^ Items adapted from Echeverri et al. ^16^

^d^ Items adapted from LaFromboise et al. ^17^

### Content Validity

A panel of experts and focus groups evaluated the initial 31 items with health care workers. Seven experts in different areas (CC in health, instrument construction and clinical care) participated in it. Each one evaluated the relevance of the 31 initial items using a 3-point Likert-type scale (1=”essential”; 2=”useful but not essential” and 3=”unnecessary”). With this information, the content validity coefficient (CVI) proposed by Lawshe was calculated. It was considered that an item should be retained when the CVI value was ≥ 0.62 ^18^ .

Four focus groups were organized with health care workers to find out their perceptions about the ownership and understanding of the items. Table 2 shows the characteristics of the participants. Each focus group was recorded, transcribed and subjected to thematic analysis. The health care workers referred to aspects of clarity, comprehensiveness and relevance of the questions, which led to modifications to the items in the instrument.

**Table 2:** Sociodemographic characteristics of participants, Santiago, Chile 2018.

	Qualitative phase		Sample Factor Analysis					Sample bias analysis				
	Focus groups		Confirmatory		Exploratory		p	Nurses		Physicians		p
	(n=29)		(n=247)		(n=236)			(n=116)		(n=217)		
	%	n	%	n	%	n		%	n	%	n	
Sex							0.18					< 0.01
Male	28	8	24	60	30	70		54.3	63	17.5	8	
Female	72	21	76	187	70	166		45.7	53	82.5	179	
Age (average)	37.2		35		35			37		34		
Occupation							0.92					
Physician	17	5	25	61	23	54		-	-	-	-	
Nurse	28	8	43	106	47	111		-	-	-	-	
Kinesiologist	7	2	10	25	9	21		-	-	-	-	
midwife	7	2	14	35	13	30		-	-	-	-	
Nutritionist	14	4	8	20	8	19		-	-	-	-	
Other	28	8		-		-		-	-	-	-	
Contact with patients							0.78					0.38
All the time	-	-	90	221	91	211		94.4	201	96.6	112	
Half or less	-	-	10	24	9	21		5.6	12	3.4	4	
Level of care												
Primary	-	-	24	58	31	72	0.09	17.8	38	27	31	0.05
Secondary	-	-	11	27	13	31	0.48	7	15	28.7	33	< 0.01
Tertiary	-	-	69	167	61	142	0.07	71.4	152	71.3	82	0.99
Graduate							0.63					
Yes	-	-	64	157	61	145		46.1	100	79.3	92	< 0.01

p corresponds to chi-square differences for the proportions and t-test for continuous variables.

Of the 31 items initially developed, nine were eliminated. The preliminary version consisted of 22 items and a 5-point Likert answer scale (1 = totally disagree, 2 = disagree, 3 = neither agree nor disagree, 4 = agree, 5 = totally agree).

### Validity based on internal structure

The analysis of the internal structure provides empirical evidence of the way in which the different items are grouped according to the theoretical dimensions proposed ^13^ . For this purpose, exploratory (EFA) and confirmatory (CFA) factor analyses were performed.

A sample of 483 health care workers was collected. The inclusion criteria was the direct contact with users. The total sample was randomized into two subsamples ( Table 2 ). In one of them (n = 236) EFA was performed and in the other (n = 247) CFA. The sufficiency of the sample size for the different analyses was checked by means of Monte Carlo simulation ^19^ , which showed that both were adequate to achieve a power of 0.80 and type error 1 < 0.05.

The KMO and Bartlett of sphericity test was performed to assess whether the data were susceptible to exploratory factor analysis (EFA) ^20^ . EFA were calculated using Weight Least Squeare Mean and Variance Adjusted estimator and Varimax rotation. The number of factors was determined considering ^20^ : (i) Kaiser’s criterion (eigenvalue >1); (ii) Scree plot; (iii) theoretical sense of the factor solution. A factor load ≥ 0.3 was considered appropriate and those items with loads ≥ 0.4 on two or more factors were eliminated ^20^ .

For confirmatory factor analysis (CFA), the factor structure obtained by EFA was considered. The model fit was evaluated according to different goodness-of-fit indexes: Root mean square error approximation (RMSEA), Standardized root mean square residuals (SRMR), Tucker Lewis index (TLI) and Comparative fit index (CFI). A value of ≥ 0.90 for the CFI and TLI ^19^ was accepted as a good fit. For the RMSEA, a value close to 0.06 ^19^ was considered and for the WRMR, < 1.0 ^21^ . Since the Chi-square index is susceptible to sample size, the ratio between the model’s chi-square and its degrees of freedom was used. Values < 3.0 suggest an acceptable fit ^22^ .

### Reliability

Reliability was determined using Cronbach’s alpha coefficient and calculated for the total scale, for each of its dimensions, and the variations of the coefficient when eliminating items were analyzed. An alpha coefficient value of 0.7 was considered.

### Bias analysis

Given the potential subjectivity of self-report scales, it is relevant to assess whether different groups of respondents understand in the same way both the questions and the response scales, as this can be a cause of measurement bias. Given the differences in the sample size of the different groups of professionals, only the presence of bias was examined among the largest groups: physicians (n = 116) and nurses (n = 217), using Differential Item Functioning Analysis (DIF), and Factor Invariance Analysis (FIA).

The presence of DIF was determined using a hierarchical ordinal regression model ^23^ . The dependent variable was the response to each of the items and the independent variables were the membership group and the total score obtained in the instrument (model 1: total score; model 2: membership group is added: model 3 is the interaction between total score and group). It was stated that there was a bias if: (i) the difference in the fit statistic (chi-square) between models 1 and 3 was significant (p < 0.01) and (ii) the explained percentage variance variation was > 13.0% between the same models.

The FIA allows determining if the factor structure of the questionnaire is equivalent among the stakeholders (examples: physicians and nurses). To determine the presence of invariance, four successive factor models were estimated (configural, metric, scalar and strict) and compared sequentially with each other (metric v/s configural, scalar v/s metric, etc.) using the CFI ^24^ . A new level of invariance was accepted if the difference in CFI in the different comparisons was < 0.01 ^25^ . A scalar level of invariance is expected to affirm that the factor structure is equivalent.

To facilitate interpretation, the scores of each of the three subscales and the total instrument were scaled to a metric between 0 and 100. Descriptive analyses of the scores obtained were performed. Mann-Whitney U and ANOVA were used for subgroup analysis. Estimates were made in Mplus 8 and SPSS v21.

This study is part of the FONIS SA16I0182 project, approved by the Ethics Committee of the South East Metropolitan Health Service in Santiago, Chile, by resolution in July 2017.

## RESULTS

Data were adequate for factor analysis (KMO = 0.824; Bartlet p < 0.01). The EFA showed that the initial 22 items were grouped into the three proposed theoretical dimensions. When analyzing the reliability for each of the dimensions, two items were identified as negatively affecting the reliability, which, after evaluating their content, were eliminated. The same situation was applied to another item that presented a load > 0.4 on two factors. A new factor analysis with 19 items successfully reproduced three dimensions, explaining 50.8% of the variance ( Table 3 ).

**Table 3:** Results of the Exploratory (EFA) and Confirmatory (CFA) Factor Analysis of the EMCC-14, Santiago, Chile 2018.

	Factor Loads EFA (n = 236)			Factor Loads CFA(n = 247)		
	Sensibility	Knowledge	Skills	Sensibility	Knowledge	Skills
Item 1	**0.445**	0.331	0.075	-		
Item 2	**0.400**	0.100	0.321	0.649		
Item 3	**0.469**	0.036	0.086	0.521		
Item 4	**0.793**	0.005	0.025	0.69		
Item 5	**0.734**	0.014	0.101	0.607		
Item 6	**0.583**	0.369	0.164			
Item 7	0.131	0.330	**0.507**		-	
Item 8	0.090	0.121	**0.894**		0.831	
Item 9	0.078	0.211	**0.742**		0.722	
Item 10	0.123	0.378	**0.524**		0.614	
Item 11	0.014	0.376	**0.579**		0.774	
Item 12	0.034	**0.574**	0.278			0.637
Item 13	0.151	**0.498**	0.262			-
Item 14	0.054	**0.572**	0.121			0.566
Item 15	0.099	**0.769**	0.228			0.732
Item 16	0.077	**0.569**	0.093			-
Item 17	0.034	**0.535**	0.169			0.474
Item 18	0.066	**0.476**	0.084			0.516
Item 19	0.183	**0.600**	0.257			0.649

The variance explained by the Exploratory Factor Analysis was 50.8%. The proper values for the factors were: sensitivity: 5.44; knowledge: 2.77; skills: 1.45. The goodness-of-fit indices for Confirmatory Factor Analysis (CFA) were: χ2(74 df) = 150.67 (p <0.01), CFI: 0.95, TLI: 0.94, RMSEA 0.065; WRMR:0.95. The correlation between factors in the CFA was: sensitivity – knowledge: r = 0.35 (p < 0.01), sensitivity – skills: r = -0.09 (p = 0.25), knowledge – skills: r = 0.73 (p < 0.01). P: question

The CFA with 19 items and three factors did not show a satisfactory adjustment (RMSEA = 0.09; CFI = 0.860; TLI = 0.839). Six items were eliminated using the modification rates ^19^ . The model with 14 items and three dimensions showed a good fit (χ *2/df* =2.03, CFI: 0.95, TLI: 0.94, RMSEA 0.065; WRMR: 0.95). The correlation between sensitivity to own prejudices and the ability to incorporate cultural aspects of users into care was not significant. The other correlations between factors were moderate ( Table 3 ).

The alpha coefficient for the overall scale was 0.7. For the dimensions of sensitivity, knowledge and skills, it was 0.65, 0.81 and 0.68 respectively.

In the DIF analysis, two items were identified that could be interpreted as biased (items 9 and 10). However, the difference in percentage variance explained between models was < 13.0% in both cases. Therefore, none of these items met sufficient statistical criteria to claim that they were biased.

In the FIA, the EMCC-14 presented configural, metric and scalar invariance ( Table 4 ). The scale maintained the same number of dimensions in both groups (configural invariance); in addition, the factor loads (metric invariance) and the item means (scalar invariance) were comparable between the groups. This suggests that the scores of physicians and nurses can be validly purchased from each other.

**Table 4:** EMCC-14 invariance analysis, Santiago, Chile 2018.

Level	χ ^2^	df	p	CFI	TLI	RMSEA	Δ CFI
Configural	186.1	148	0.00	0.98	0.975	0.039	
Metric	188.5	159	0.05	0.984	0.982	0.033	0.004
Scale	196.6	170	0.08	0.986	0.985	0.031	0.002

χ ^2^: Chi-square of the factor model; df: degrees of freedom of the factor model; CFI: Comparative fit index; TLI: Tucker Lewis index; RMSEA: Root mean square error approximation; Δ CFI: Difference of CFI between models

The descriptive analysis of the items of the EMCC-14 is found in Table 1 . The average CC score achieved in the total sample was 74.6 points (sd = 10). The dimension with the highest score was knowledge (M = 85.6, sd = 12.6), followed by skills (M = 77.7, sd = 12.7) and sensitivity to own prejudices (M = 58, sd = 22.3). This pattern was maintained by stratifying according to sex, profession, level of the health system in which they work and degree of contact with patients ( Table 5 ). Women presented lower sensitivity scores (p < 0.01), while their scores were higher in knowledge and skills (p < 0.01). Differences were also observed in the level of CC according to the participants’ profession. Physicians achieved higher levels than midwives (p < 0.001) and nutritionists (p = 0.007) in sensitivity, while scores for nutritionists were higher than for physicians in knowledge (p = 0.001).

**Table 5:** Average for the level of general cultural competence and each dimension in different subgroups, Santiago Chile 2018.

	Sensitivity (sd)	p	Knowledge (sd)	p	Skills (sd)	p	Cultural competence total (sd)	p
Sex								
Male	62.5 (20.9)	< 0.01	82.9 (13.2)	< 0.01	74.7 (11.9)	< 0.01	73.7 (9.6)	0.31
Female	56.3 (22.6)		86.6 (12.2)		78.8 (12.8)		74.9 (10.1)	
Age (average)								
Occupation								
Physician	65.1 (21.5)	< 0.01	82.8 (13.2)	< 0.01	76.8 (11.5)	0.06	75.2 (9.7)	0.3
Nurse	57.4 (22.3)		86.2 (12.4)		76.8 (13.4)		74.3 (10.4)	
Kinesiologist	60.1 (19.6)		84.7 (12.8)		79.5 (10.5)		75.5 (9.4)	
midwife	49.2 (22.2)		85.8 (12.7)		78 (13.8)		72.4 (9.8)	
Nutritionist	51.3 (21.5)		91.8 (8.6)		83 (10.9)		76.4 (9.2)	
Contact with patients								
All the time	58.3 (22.4)	0.2	85.3 (12.6)	0.04	77.8 (12.7)	0.65	74.6 (9.9)	0.78
Half or less	54.3 (21.2)		89.3 (11.8)		76.7 (12.2)		73.7 (11)	
Level of care								
Primary/ Secondary	58.8 (22.6)	0.54	86.7 (12.1)	0.23	78.9 (12.3)	0.14	75.5 (10.2)	0.08
Tertiary	57.8 (22.3)		85.2 (12.8)		77 (13.1)		74.2 (9.9)	

*sd* : standard deviation

## DISCUSSION

This study reports on the development and validation of an instrument to measure cultural competence in health care workers in Chile (EMCC-14). The analyses support the validity, reliability and high comparability between the groups with greater participation in the sample (physicians and nurses) of the EMCC-14.

Most existing questionnaires for measuring CC provide some evidence of content validity. However, many of them do not have appropriate psychometric analyses ^10 , 11^ . The EMCC-14 provides both aspects, in addition to having questions that are representative of the three dimensions of CC (sensitivity, knowledge and skills ^8^ .

Moreover, based on the CFA, evidence of discriminatory validity was provided. The three measured dimensions constitute different but complementary constructs, which is reflected in the fact that the correlation between the different factors is adequate and that each of the items is exclusively related to a theoretical dimension. In the analysis of correlation between factors, an association was found between sensitivity and knowledge, and between knowledge and skills. This could indicate that working on sensitivity to one’s own prejudices would be a good precursor to CC, as has been proposed in the literature ^26^ . In addition to the above, the empirical distinction reached of the constructs of sensitivity, knowledge and skills distinguishes this study from others previously published ^27^ , in which a greater number of dimensions are presented and the constructs that these represent tend to overlap.

The EMCC-14 shows adequate internal consistency. However, two of the subscales obtained a low reliability of 0.7, which could be explained by the small number of items in each of them. Despite this, the indices obtained are comparable with those of other instruments in this area ^27^ .

Most CC instruments have been validated in a single group of health care providers ^10 , 28^ . This study, in addition to including a diverse sample of professionals, evaluated the statistical presence of bias among the groups with greatest representation in our sample (physicians and nurses). These analyses suggest that both the questions and the response scales of the EMCC-14 can be interpreted in an equivalent manner in both groups and, therefore, their results are comparable.

A relevant result is the low score achieved by all professionals in the sensitivity dimension. Sensitivity implies being aware of our own prejudices and preconceived notions ^8 , 28^ . Our prejudices as well as stereotypes towards certain cultural groups are closely related to the way we relate to these groups ^11^ .

Improving the sensitivity of professionals is key to achieving CC. Majumdar, Browne, Roberts and Carpio ^29^ showed that a training program for health professionals focused on this area, in addition to favoring the development of sensitivity, could positively affect knowledge and skills and favorably impact user satisfaction ^29^ . This is consistent with our other findings suggesting that increased sensitivity may facilitate knowledge acquisition. In turn, greater knowledge would precede greater skills in delivering culturally competent health care.

This is the first instrument to measure CC developed in Chile and represents an advance in this issue in the country. One of the strengths of this study is the inclusion of a diverse sample of health providers, from different levels of health care.

In addition, this study provides a benchmark of the level of health care worker’s CC, which is a good starting point for intervention, either by developing CC training or adapting programs that have been shown to be effective, which can address the areas of greatest deficit. In addition, the EMCC-14 could be used in the field of research on this topic, thus contributing to addressing health inequalities.

Chile has a mixed health system made up of public and private providers. This study only considered a sample of public sector workers of areas of high social vulnerability. Despite this, CC is a new issue in Chile, and it is possible that there are other variables more relevant than administrative dependence that may influence its development. Examples of this are the level of contact of professionals with different populations or individual variables (such as personality characteristics).

In addition to the evidence of the validity and reliability of the EMCC-14 provided in this study, the relationship of the scores of this instrument with other variables ^13^ such as user satisfaction or trust in health care providers could also be explored. This could provide evidence for the predictive validity of EMCC. The limitations noted do not affect the validity of the results of this study.

EMCC is an instrument that has shown favorable evidence about its validity and reliability to measure the level of CC in different health care workers in Chile. Its availability is transformed into a contribution to the health care, academic training and research fields.
