# Supplementary material for: Development of an instrument to measure the cultural competence of health care workers
Source: Rev Saude Publica. 2020 Mar 12;54:29. doi: 10.11606/s1518-8787.2020054001695 (PMC7069710; doi:10.11606/s1518-8787.2020054001695)
Supplement: Translation

# Translation: Desarrollo de un instrumento para medir competencia cultural en trabajadores de Salud

**Authors:** Victor Pedrero, Margarita Bernales, Macarena Chepo, Jorge Manzi, Miguel Pérez, Paulina Fernández

**Affiliations:** I Chile Universidad Andrés Bello. Facultad de Enfermería, Santiago, Chile; II Chile Pontificia Universidad Católica de Chile. Facultad de Medicina, Escuela de Enfermería, Programa de Salud Global, Santiago, Chile; III Chile Pontificia Universidad Católica de Chile. Facultad de Ciencias Sociales. Escuela de Psicología, Santiago, Chile; IV Estados Unidos California State University Fresno. College of Health and Human Services. Department of Public Health, California, Estados Unidos; V Psychologist. Independent consultant.

**Keywords:** Competencia Cultural, Asistencia Sanitaria Culturalmente Competente, Conocimientos, Actitudes y Práctica en Salud, Estudios de Validación

## Abstract

**OBJETIVO:**

Validar un instrumento de medición de competencia cultural en trabajadores de salud de Chile.

**MÉTODOS:**

Utilizando el modelo teórico de Sue y Sue, se diseñó un instrumento de medición el cual fue evaluado por trabajadores de salud y expertos. Este instrumento se aplicó a una muestra diversa de 483 proveedores de salud, durante 2018 en Santiago de Chile. Se realizó análisis factorial exploratorio, confirmatorio, estimación de confiabilidad y análisis de sesgo de medición. Se estimó el nivel de competencia cultural alcanzado por los profesionales.

**RESULTADOS:**

El instrumento final contó con 14 ítems los cuales se agruparon en tres dimensiones: sensibilidad a los propios prejuicios, conocimiento cultural y habilidades para trabajar en entornos culturalmente diversos. Esta herramienta mostró buen ajuste en los modelos factoriales, adecuada confiabilidad y ausencia de evidencias de sesgo de medición. Los trabajadores de salud evaluados exhibieron un bajo nivel de sensibilidad a los propios prejuicios en comparación con las otras dimensiones evaluadas.

**CONCLUSIONE:**

La Escala de Medición de Competencia Cultural en trabajadores de salud (EMCC-14) es una herramienta confiable, con soporte inicial para su validez, que puede usarse en el contexto Chileno. Además, los resultados de este estudio podrían guiar algunas posibles intervenciones en el sector de la salud para fortalecer el nivel de competencia cultural.

## INTRODUCCIÓN

Chile se ha transformado progresivamente en un país más diverso. El 4,4% de la población es migrante internacional^1^ , un 12,8% declara pertenecer a algún pueblo indígena^1^ y el 3,1% de los hombres y 2,3% de las mujeres se identifican con un género distinto a su sexo de nacimiento^2^ . En el ámbito sanitario, Chile ha comprometido la entrega de cuidados de salud centrados en los pacientes^3^ . Esto significa, entre otras cosas, atender necesidades particulares de diferentes grupos de la población y brindar un trato libre de discriminación. Para lograr esto, es importante considerar el rol de los aspectos culturales en la relación de las personas con los procesos de salud y enfermedad^3^ .

La cultura corresponde a un sistema de marcos conceptuales y esquemas compartidos, utilizados por los miembros de un grupo (o subgrupo) para interpretar la realidad y relacionarse con el mundo que los rodea, incluida su salud^4^ . Cuando los aspectos culturales no son considerados en la atención de salud, las interacciones entre pacientes y profesionales son menos centradas en el paciente, más breves, y menos positivas^5^ . La competencia cultural (CC) en salud se refiere a la reflexión y cuestionamiento permanentes por parte del trabajador de la salud en torno a cómo la cultura -propia y del paciente- impacta en la interacción con los usuarios del sistema de salud^6^ . CC incluye el compromiso de brindar atención de salud que tenga en cuenta las creencias y comportamientos del paciente.

Pese a que existen diferentes definiciones de CC, hay consenso en que es un constructo multidimensional (incluye al menos tres áreas: sensibilidad, habilidades y conocimiento) y multinivel (involucra tanto los profesionales de salud como sistemas de salud)^7 , 8^ . Sue y Sue^8^ proponen un marco conceptual para CC que recoge estos dos aspectos, donde se plantean cuatro niveles de competencia cultural: individual (profesionales de salud), profesional (prácticas profesionales), organizacional (prácticas y políticas institucionales) y social (políticas sociales). En cada uno de estos niveles, se debe considerar: la sensibilidad a los prejuicios, el conocimiento de los aspectos culturales y la habilidad para integrar tales aspectos en la atención.

Una de las características relevantes de la CC es que puede ser entrenada^9^ . Sin embargo, su medición sigue siendo un desafío, lo cual dificulta evaluar la efectividad de las intervenciones en esta línea^9^ . Existen varias escalas de medición de CC, sin embargo se han cuestionado aspectos asociados a su validez^10 , 11^ . Por otra parte, varios instrumentos habitualmente remarcan la relevancia de aspectos étnicos y raciales por sobre otros encuentros culturales, lo cual podría dificultar su utilización en otros contextos donde dichos factores no explican de forma predominante la diversidad cultural^12^ .

El presente estudio tuvo por objetivo validar un instrumento de medición de CC en Chile.

## MÉTODOS

El desarrollo de la Escala de Medición de CC en trabajadores de salud en Chile (EMCC-14) consideró^13^ : (i) desarrollo de ítems, (ii) estudio de validez de contenido; (iii) análisis de validez de estructura interna; (iii) estimación de confiabilidad y; (iv) análisis de sesgo. La recolección de datos tuvo lugar en la ciudad de Santiago de Chile durante 2018.

### Desarrollo de Ítems

Además de considerar los componentes propuestos por Sue y Sue^8^ , se revisó la literatura en el área de CC e instrumentos de medición publicados previamente. La primera versión del EMCC-14 contó con 31 ítems divididos en tres dimensiones teóricas (sensibilidad, conocimiento y habilidades)^9^ . La versión final del EMCC-14 cuenta con 14 ítems ( Tabla 1 ). De éstos, ocho fueron adaptados de instrumentos previos^14^ y seis creados por los autores.

**Tabla 1:** Escala de Medición de competencia cultural para trabajadores de salud (EMCC-14), Santiago, Chile 2018.

Pensando en su práctica clínica habitual, frente a cada pregunta, indique qué tan de acuerdo o desacuerdo se encuentra usted.	Totalmente en desacuerdo	En desacuerdo	Ni de acuerdo ni en desacuerdo	De acuerdo	Totalmente de acuerdo
	(1 punto) (%)	(2 puntos) (%)	(3 puntos) (%)	(4 puntos) (%)	(5 puntos) (%)
1. Creo que pacientes con diferentes creencias y costumbres tienen distintas expectativas y/o necesidades en la atención en salud^a^	7,1	10,2	9,6	38,1	35,0
2. Creo que las creencias, valores y costumbres de los pacientes afectan su salud^b^.	8,3	10,6	17,0	37,6	26,6
3. Creo que mi contexto cultural influye sobre mis actitudes y creencias acerca de otros grupos culturales^c^.	18,9	21,8	15,6	30,9	12,9
4. Soy consciente de que mis creencias acerca de los pacientes influyen en las recomendaciones terapéuticas que les entrego.	20,8	23,1	18,3	28,5	9,2
5. Las creencias, valores y costumbres de los pacientes deben ser valorados en la atención en salud.	0,2	0,6	2,9	33,2	63,1
6. Considero que conocer mejor las creencias y costumbres de los pacientes me ayuda a plantear un tratamiento más adecuado.	0,8	1,0	7,9	36,4	53,8
7. Considero que cada paciente tiene su propio concepto de salud y enfermedad.	0,2	1,9	7,1	45,0	45,9
8. Considero que los problemas de salud del paciente deben ser comprendidos dentro de su contexto cultural (creencias, valores y costumbres)^d^.	0,2	1,1	6,6	48,2	44,0
9. Les pido al paciente y a su familia que manifiesten las expectativas que tienen con respecto al cuidado y atención en salud^b^.	1,0	6,6	18,0	46,7	27,6
10. Soy capaz de reconocer barreras potenciales para acceder a servicios de salud a las que se pueden ver enfrentados los diferentes pacientes^b^.	0,4	2,1	7,0	61,9	28,5
11. Soy capaz de establecer metas y/o objetivos terapéuticos considerando el contexto cultural (creencias y costumbres) de mis pacientes y sus necesidades^c^.	0,6	1,5	14,9	51,0	32,0
12. Registro en la ficha clínica los datos sobre creencias y costumbres recogidos en la valoración del paciente.	4,9	11,1	21,1	38,9	24,0
13. Me esfuerzo por explicar al paciente su tratamiento médico, incluso si él cree que la causa de su enfermedad es sobrenatural.	0,2	1,1	5,9	44,4	48,4
14. Estoy atento a posibles dificultades que puedan surgir durante la atención en salud debido a las diferencias culturales entre el paciente y yo.	0,2	1,1	4,5	49,9	44,3

^a^ítems adaptados de Cai et al^14^

^b^Ítems adaptados de Doorenbos et al^15^

^c^Ítems adaptados de Echeverri et al^16^

^d^ítems adaptados de LaFromboise et al^17^

### Validez de Contenido

Los 31 ítems iniciales fueron sometidos a evaluación por un panel de expertos y grupos focales con trabajadores de salud. Participaron del panel de expertos siete expertos en diferentes áreas (CC en salud, construcción de instrumentos y atención clínica). Cada uno evaluó la pertinencia de los 31 ítems iniciales mediante una escala tipo Likert de 3 puntos (1= “esencial”; 2= “útil pero no esencial” y 3= “innecesario”). Con esta información, se estimó el coeficiente de validez de contenido (CVI) propuesto por Lawshe. Se consideró que un ítem debía ser retenido cuando el valor del CVI fuese ≥ 0,62^18^ .

Se realizaron cuatro grupos focales con trabajadores de salud para conocer sus percepciones sobre la pertenencia y comprensión de los ítems. Las características de los participantes están en la Tabla 2 . Cada grupo focal fue grabado, transcrito y sometido a análisis temático. Los trabajadores de salud aludieron a aspectos de claridad, extensión y pertinencia de las preguntas, con lo cual se realizaron modificaciones a los ítems del instrumento.

**Tabla 2:** Características Sociodemográficas de los participantes, Santiago, Chile 2018.

	Fase cualitativa		Muestra Análisis Factorial					Muestra análisis de sesgo				
	Grupos focales		Confirmatorio		Exploratorio		p	Enfermeros		Médicos		p
	(n=29)		(n=247)		(n=236)			(n=116)		(n=217)		
	%	n	%	n	%	n		%	n	%	n	
**Género**							0,18					< 0,01
Masculino	28	8	24	60	30	70		54,3	63	17,5	8	
Femenino	72	21	76	187	70	166		45,7	53	82,5	179	
**Edad (media)**	37,2		35		35			37		34		
**Profesión**							0,92					
Médico	17	5	25	61	23	54		-	-	-	-	
Enfermero	28	8	43	106	47	111		-	-	-	-	
Kinesiólogo	7	2	10	25	9	21		-	-	-	-	
Matrona	7	2	14	35	13	30		-	-	-	-	
Nutricionista	14	4	8	20	8	19		-	-	-	-	
Otro	28	8		-		-		-	-	-	-	
**Contacto con pacientes**							0,78					0,38
Todo el tiempo	-	-	90	221	91	211		94,4	201	96,6	112	
La mitad o menos	-	-	10	24	9	21		5,6	12	3,4	4	
**Nivel de atención**												
Primario	-	-	24	58	31	72	0,09	17,8	38	27	31	0,05
Secundario	-	-	11	27	13	31	0,48	7	15	28,7	33	< 0,01
Terciario	-	-	69	167	61	142	0,07	71,4	152	71,3	82	0,99
**Posgrado**							0,63					
Si	-	-	64	157	61	145		46,1	100	79,3	92	< 0,01

El p corresponde a diferencias de chi cuadrado para las proporciones y prueba t para variables continuas.

De los 31 ítems inicialmente desarrollados, nueve fueron eliminados. La versión preliminar quedó conformada por 22 ítems y una escala de respuesta tipo Likert de 5 puntos (1 = totalmente en desacuerdo, 2 = en desacuerdo, 3 = ni de acuerdo ni en desacuerdo, 4 = de acuerdo, 5 = totalmente de acuerdo).

### Validez basada en la estructura interna

El análisis de la estructura interna aporta evidencia empírica de la forma en que se agrupan los diferentes ítems de acuerdo a las dimensiones teóricas propuestas^13^ . Para esto, se realizó análisis factorial exploratorio (AFE) y confirmatorio (AFC).

Se recolectó una muestra a conveniencia de 483 trabajadores de salud. El criterio de inclusión fue que estos tuviesen contacto directo con usuarios. La muestra total fue aleatorizada en dos submuestras ( Tabla 2 ). En una de ellas (n = 236) se llevó a cabo AFE y en la otra (n = 247) AFC. Se comprobó la suficiencia del tamaño muestral para los diferentes análisis mediante simulación de Montecarlo^19^ , la cual mostró que ambas eran adecuadas para alcanzar un poder de 0,80 y error tipo 1 < 0,05.

Se realizó la prueba de esfericidad de Bartlett y KMO para evaluar si los datos eran susceptibles de ser sometidos a análisis factorial exploratorio (EFA)^20^ . Se estimó EFA utilizando Mínimos Cuadrados Ponderados por Media y Varianza y rotación Varimax. El número de factores fue determinado considerando^20^ : (i) criterio de Kaiser (eigen value > 1): (ii) análisis del grafico de sedimentación; (iii) sentido teórico de la solución factorial. Se consideró como apropiado una carga factorial ≥ 0,3 y se eliminaron aquellos ítems con cargas ≥ 0,4 en dos o más factores^20^ .

Para el análisis factorial confirmatorio *(* AFC) se consideró la estructura factorial obtenida mediante EFA. El ajuste del modelo se evaluó de acuerdo a diferentes índices de bondad de ajuste: *Root mean square error aproximation* (RMSEA), *Standarized root mean square residuals* (SRMR), *Tucker Lewis index* (TLI) y *Comparative fit index* (CFI). Se aceptó como buen ajuste un valor ≥ 0,90 para el CFI y TLI^19^ . Para el RMSEA, se consideró un valor cercano a 0,06^19^ y para el WRMR, < 1,0^21^ . Dado que el índice de Chi cuadrado es sensible al tamaño muestral, se utilizó la razón entre el chi cuadrado del modelo y sus grados de libertad. Valores < 3,0 sugieren un ajuste aceptable^22^ .

### Confiabilidad

La confiabilidad fue determinada utilizando coeficiente alpha de Cronbach. Se estimó la confiabilidad para la escala total, para cada una de sus dimensiones y se analizaron las variaciones del coeficiente al eliminar ítems. Se consideró como referencia un valor de coeficiente alpha de 0,7.

### Análisis de sesgo

Dada la potencial subjetividad de escalas de autorreporte, es relevante evaluar si los diferentes grupos de examinados comprenden de la misma forma tanto las preguntas como las escalas de respuesta, ya que esto puede ser una fuente de sesgo de medición. Atendiendo a las diferencias en el tamaño muestral de los diferentes grupos de profesionales, solo se examinó la presencia de sesgo entre los grupos de mayor tamaño: médicos (n = 116) y enfermeras (n = 217), utilizando Análisis de Funcionamiento Diferencial de ítems (DIF) y Análisis de Invarianza Factorial (AIF).

La presencia de DIF se determinó utilizando un modelo jerárquico de regresión ordinal^23^ . La variable dependiente fue la respuesta a cada uno de los ítems y las variables independientes fueron el grupo de pertenencia y el puntaje total obtenido en el instrumento (modelo 1: puntaje total; modelo 2: se agrega grupo de pertenencia: modelo 3 se agrega la interacción entre puntaje total y grupo). Se afirmó que existía sesgo si: (i) la diferencia en el estadístico de ajuste (chi cuadrado) entre los modelos 1 y 3 era significativa (p < 0,01) y (ii) la variación del porcentaje de varianza explicada era > 13,0% entre los mismos modelos.

El AIF permite determinar si la estructura factorial de cuestionario es equivalente entre los grupos de interés (ejemplos médicos y enfermeras). Para determinar la presencia de invarianza, se estimaron cuatro modelos factoriales sucesivos (configural, métrico, escalar y estricto) y se compararon secuencialmente entre sí (métrico v/s configural, escalar v/s métrico, etc.) utilizando el CFI^24^ . Se aceptó un nuevo nivel de invarianza si la diferencia de CFI en las distintas comparaciones era < 0,01^25^ . se espera un nivel de invarianza escalar para afirmar que la estructura es equivalente.

Para facilitar la interpretación, se escalaron los puntajes de cada una de las tres subescalas y del instrumento total a una métrica entre 0 y 100. Se realizaron análisis descriptivos de los puntajes obtenidos. Se utilizó U de Mann-Whitney y ANOVA para el análisis de subgrupos. Las estimaciones se realizaron en Mplus 8 y SPSS v21.

Este trabajo es parte del proyecto FONIS SA16I0182, el cual fue aprobado por el Comité de ética del Servicio de Salud Metropolitano Sur Oriente en Santiago de Chile, mediante resolución de Julio de 2017.

## RESULTADOS

Los datos resultaron adecuados para realizar análisis factorial (KMO = 0,824; Bartlet p < 0,01). El AFE mostró que los 22 ítems iniciales se agruparon en las tres dimensiones teóricas propuestas. Al analizar la confiabilidad para cada una de las dimensiones, se identificaron dos ítems que afectaban negativamente la confiabilidad, los cuales, tras evaluar su contenido, fueron eliminados. La misma situación se aplicó a otro ítem que presentó una carga > 0,4 en dos factores. Un nuevo análisis factorial con 19 ítems, reprodujo exitosamente tres dimensiones, explicando el 50,8% de la varianza ( Tabla 3 ).

**Tabla 3:** Resultados del Análisis Factorial Exploratorio (AFE) y Confirmatorio (AFC) del EMCC-14, Santiago, Chile 2018.

	Cargas factoriales AFE (n = 236)			Cargas factoriales AFC(n = 247)		
	Sensibilidad	Conocimiento	Habilidades	Sensibilidad	Conocimiento	Habilidades
Ítem 1	**0,445**	0,331	0,075	-		
Ítem 2	**0,400**	0,100	0,321	0,649		
Ítem 3	**0,469**	0,036	0,086	0,521		
Ítem 4	**0,793**	0,005	0,025	0,69		
Ítem 5	**0,734**	0,014	0,101	0,607		
Ítem 6	**0,583**	0,369	0,164	-		
Ítem 7	0,131	0,330	**0,507**		-	
Ítem 8	0,090	0,121	**0,894**		0,831	
Ítem 9	0,078	0,211	**0,742**		0,722	
Ítem 10	0,123	0,378	**0,524**		0,614	
Ítem 11	0,014	0,376	**0,579**		0,774	
Ítem 12	0,034	**0,574**	0,278			0,637
Ítem 13	0,151	**0,498**	0,262			-
Ítem 14	0,054	**0,572**	0,121			0,566
Ítem 15	0,099	**0,769**	0,228			0,732
Ítem 16	0,077	**0,569**	0,093			-
Ítem 17	0,034	**0,535**	0,169			0,474
Ítem 18	0,066	**0,476**	0,084			0,516
Ítem 19	0,183	**0,600**	0,257			0,649

La varianza explicada por el Análisis Factorial Exploratorio fue 50,8%. Los valores propios para los factores fueron: sensibilidad: 5,44; conocimiento: 2,77; habilidades: 1,45. Los índices de bondad de ajuste para el Análisis Factorial Confirmatorio (AFC) fueron: χ^2^_(74 df)_= 150,67 (p <0,01), CFI: 0,95, TLI: 0,94, RMSEA 0,065; WRMR:0,95. La correlación entre factores en el AFC fue: sensibilidad – conocimiento: r = 0,35 (p < 0,01), sensibilidad-habilidades: r = -0,09 (p = 0,25), conocimiento-habilidades: r = 0,73 (p < 0,01). P: pregunta

El AFC con 19 ítems y tres factores no mostró un ajuste satisfactorio (RMSEA = 0,09; CFI = 0,860; TLI = 0,839). Utilizando los índices de modificación^19^ , se eliminaron seis ítems. El modelo con 14 ítems y tres dimensiones mostró un buen ajuste (χ^2^ */df =2,03,* CFI: 0,95, TLI: 0,94, RMSEA 0,065; WRMR:0,95). La correlación entre los factores no fue significativa para la relación entre sensibilidad a los propios prejuicios y las habilidades para incorporar aspectos culturales de los usuarios en la atención. Las otras correlaciones entre factores fueron moderadas ( Tabla 3 ).

El coeficiente alpha para la escala global fue de 0,7. Para las dimensiones de sensibilidad, conocimiento y habilidades fue 0,65, 0,81 y 0,68 respectivamente.

En el análisis de DIF, se identificaron dos ítems susceptibles de ser interpretados como sesgados (ítems 9 y 10). Sin embargo, la diferencia en el porcentaje de varianza explicada entre modelos fue < 13,0% en ambos casos. Por lo tanto, ninguno de estos ítems reunió criterios estadísticos suficientes para afirmar que se encontraban sesgado.

En el AIF la EMCC-14 presentó invarianza configural, métrica y escalar ( Tabla 4 ). Es decir, la escala mantuvo el mismo número de dimensiones en ambos grupos (invarianza configural), además, las cargas factoriales (invarianza métrica) y las medias de los ítems (invarianza escalar) fueron comparables entre los grupos. Esto sugiere que los puntajes de médicos y enfermeras pueden ser comprados entre si de forma válida.

**Tabla 4:** Análisis de invarianza del EMCC-14, Santiago, Chile 2018.

Nivel	χ^2^	df	p	CFI	TLI	RMSEA	Δ CFI
Configural	186,1	148	0,00	0,98	0,975	0,039	
Métrica	188,5	159	0,05	0,984	0,982	0,033	0,004
Escalar	196,6	170	0,08	0,986	0,985	0,031	0,002

χ^2^: Chi cuadrado del modelo factorial; df: grados de libertad del modelo factorial; *CFI* : Comparative fit index; *TLI* : Tucker Lewis index; *RMSEA* : Root mean square error aproximation; Δ CFI: Diferencia de CFI entre modelos

El análisis descriptivo de los ítems del EMCC-14 se encuentra en la Tabla 1 . El puntaje promedio de CC alcanzado en el total de la muestra fue 74,6 puntos (de = 10). La dimensión con el puntaje más alto fue conocimiento (M = 85,6, de = 12,6), seguido de habilidades (M = 77,7, de = 12,7) y sensibilidad a los propios prejuicios (M = 58, de = 22,3). Este patrón se mantuvo al estratificar según sexo, profesión, nivel del sistema de salud en que se desempeña y grado de contacto con pacientes ( Tabla 5 ). Las mujeres presentaron puntajes menores de sensibilidad (p < 0,01), mientras que sus puntajes fueron mayores en conocimiento y habilidades (p < 0,01). También se observaron diferencias en el nivel de CC según la profesión de los participantes. Los médicos alcanzaron niveles más altos que matronas (p < 0,001) y nutricionistas (p = 0,007) en sensibilidad, mientras que los puntajes para nutricionistas fueron más altos que para médicos en conocimiento (p = 0,001).

**Tabla 5:** Promedio para el nivel de competencia cultural general y cada dimensión en diferentes subgrupos, Santiago Chile 2018.

	Sensibilidad (de)	p	Conocimiento (de)	p	Habilidades (de)	p	Competencia cultural total (de)	p
**Género**								
Masculino	62,5 (20,9)	< 0,01	82,9 (13,2)	< 0,01	74,7 (11,9)	< 0,01	73,7 (9,6)	0,31
Femenino	56,3 (22,6)		86,6 (12,2)		78,8 (12,8)		74,9 (10,1)	
**Edad (media)**								
**Profesión**								
Médico	65,1 (21,5)	< 0,01	82,8 (13,2)	< 0,01	76,8 (11,5)	0,06	75,2 (9,7)	0,3
Enfermero	57,4 (22,3)		86,2 (12,4)		76,8 (13,4)		74,3 (10,4)	
Kinesiólogo	60,1 (19,6)		84,7 (12,8)		79,5 (10,5)		75,5 (9,4)	
Matrona	49,2 (22,2)		85,8 (12,7)		78 (13,8)		72,4 (9,8)	
Nutricionista	51,3 (21,5)		91,8 (8,6)		83 (10,9)		76,4 (9,2)	
**Contacto con pacientes**								
Todo el tiempo	58,3 (22,4)	0,2	85,3 (12,6)	0,04	77,8 (12,7)	0,65	74,6 (9,9)	0,78
La mitad o menos	54,3 (21,2)		89,3 (11,8)		76,7 (12,2)		73,7 (11)	
**Nivel de atención**								
Primario/Secundario	58,8 (22,6)	0,54	86,7 (12,1)	0,23	78,9 (12,3)	0,14	75,5 (10,2)	0,08
Terciario	57,8 (22,3)		85,2 (12,8)		77 (13,1)		74,2 (9,9)	

*de* : desviación estándar

## DISCUSIÓN

El presente estudio da cuenta del desarrollo y validación de un instrumento para medir competencia cultural en trabajadores de salud en Chile (EMCC-14). Los análisis apoyan la validez, confiabilidad y alta comparabilidad entre los grupos con mayor participación en la muestra (médicos y enfermeras) del EMCC-14.

La mayoría de los cuestionarios existentes para medir CC entregan alguna evidencia de validez de contenido. Sin embargo, muchos de ellos no poseen análisis psicométricos apropiados^10 , 11^ . El EMCC-14 da cuenta de ambos aspectos, sumado a contar con preguntas representativas de las tres dimensiones de la CC (sensibilidad, conocimiento y habilidades)^8^ .

Por otra parte, mediante el AFC, se aportó evidencia de validez discriminante. Las tres dimensiones medidas constituyen constructos distintos pero complementarios entre sí, lo cual se ve reflejado en que la correlación entre los diferentes factores es adecuada y en que cada uno de los ítems se relaciona exclusivamente con una dimensión teórica. En el análisis de correlación entre factores, se encontró asociación entre sensibilidad y conocimiento, y entre conocimiento y habilidades. Eso podría sugerir que trabajar sobre la sensibilidad a los propios prejuicios sería un buen precursor de CC, tal como ha sido propuesto en la literatura^26^ . Sumado a lo anterior, la distinción empírica alcanzada de los constructos de sensibilidad, conocimiento y habilidades distingue a este estudio de otros previamente publicados^27^ , donde se presenta un número mayor de dimensiones y los constructos que estas representan tienden a superponerse.

El EMCC-14 muestra una adecuada consistencia interna. Sin embargo, dos de las subescalas obtuvieron una confiabilidad ligeramente baja 0,7, lo cual podría ser explicado por el reducido número de ítems en cada una de ellas. Pese a esto, los índices obtenidos son comparables con los de otros instrumentos en esta área^27^ .

La mayoría de los instrumentos de CC han sido validados en un único grupo de proveedores de salud^10 , 28^ . Este trabajo, además de incluir una muestra diversa de profesionales, evaluó la presencia estadística de sesgo entre los grupos con mayor representación en nuestra muestra (médicos y enfermeras). Estos análisis sugieren que tanto las preguntas, como las escalas de repuesta del EMCC-14 pueden ser interpretadas de forma equivalente en ambos grupos y, por lo tanto, sus resultados son comparables.

Un resultado relevante es el bajo puntaje alcanzado por todos los profesionales en la dimensión de sensibilidad. La sensibilidad implica ser conscientes de nuestros propios prejuicios y nociones preconcebidas^8 , 28^ . Nuestros prejuicios así como los estereotipos hacia ciertos grupos culturales se relacionan estrechamente con la forma en que nos relacionamos con dichos grupos^11^ .

Mejorar la sensibilidad de los profesionales es clave para lograr CC. Majumdar, Browne, Roberts y Carpio^29^ mostraron que un programa de entrenamiento para profesionales de salud enfocado en esta área, además de favorecer el desarrollo de sensibilidad, puede afectar positivamente el conocimiento y habilidades e impactar favorablemente la satisfacción de los usuarios^29^ . Esto es concordante con otros de nuestros hallazgos que sugieren que una mayor sensibilidad podría facilitar la adquisición de conocimiento. A su vez, un mayor conocimiento precedería a mayores habilidades para entregar una atención de salud culturalmente competente.

Este es el primer instrumento para medir CC desarrollado en Chile y representa un avance en esta temática en el país. Una de las fortalezas de este estudio es la inclusión de una muestra diversa de proveedores de salud, de diferentes niveles de atención en salud.

Además, este trabajo entrega una referencia del nivel de CC de trabajadores de salud, lo que constituye un buen punto de partida para intervenir, ya sea desarrollando entrenamiento en CC o adaptando programas que han demostrado ser efectivos, que puedan abordar las áreas más deficitarias. Además, el EMCC-14 podría ser utilizado en el campo de la investigación en este tema, aportando así en el abordaje de las desigualdades en salud.

Chile posee un sistema mixto de salud conformado por prestadores públicos y privados. Este estudio solo consideró una muestra de trabajadores del sector público que se desempeñan en áreas de alta vulnerabilidad social. Pese a esto, la CC es un tema nuevo en Chile y es posible que existan otras variables más relevantes que la dependencia administrativa que puedan influir en su desarrollo. Ejemplos de esto son el nivel de contacto de los profesionales con diferentes poblaciones o variables individuales (como rasgos de personalidad).

Además de las evidencias de la validez y confiabilidad del EMCC-14 entregadas en este estudio, también podría ser explorada la relación de los puntajes de este instrumento con otras variables^13^ como satisfacción usuaria o confianza en los prestadores de salud. Esto podría entregar evidencia sobre la validez predictiva del EMCC. Las limitaciones señaladas no afectan la validez de los resultados de este estudio.

La EMCC es un instrumento que ha mostrado poseer evidencia favorable acerca de su validez y confiabilidad para medir el nivel de CC en diferentes trabajadores de salud en Chile. Su disponibilidad se transforma en una contribución en el área asistencial, formación académica e investigación.
